# Semitransparent Sb_2_S_3_ thin film solar cells by ultrasonic spray pyrolysis for use in solar windows

**DOI:** 10.3762/bjnano.10.230

**Published:** 2019-12-06

**Authors:** Jako S Eensalu, Atanas Katerski, Erki Kärber, Lothar Weinhardt, Monika Blum, Clemens Heske, Wanli Yang, Ilona Oja Acik, Malle Krunks

**Affiliations:** 1 Department of Materials and Environmental Technology, Tallinn University of Technology, Ehitajate tee 5, 19086, Estonia; 2 Department of Chemistry and Biochemistry, University of Nevada, Las Vegas (UNLV), 4505 Maryland Parkway, Las Vegas, NV 89154-4003, USA; 3 Institute for Photon Science and Synchrotron Radiation (IPS), Karlsruhe Institute of Technology (KIT), 76344 Eggenstein-Leopoldshafen, Germany; 4 Advanced Light Source, Lawrence Berkeley National Laboratory, 1 Cyclotron Road, Berkeley, California, 94720, USA

**Keywords:** antimony sulfide, semitransparent solar cells, solar windows, thin films, ultrasonic spray pyrolysis

## Abstract

The integration of photovoltaic (PV) solar energy in zero-energy buildings requires durable and efficient solar windows composed of lightweight and semitransparent thin film solar cells. Inorganic materials with a high optical absorption coefficient, such as Sb_2_S_3_ (>10^5^ cm^−1^ at 450 nm), offer semitransparency, appreciable efficiency, and long-term durability at low cost. Oxide-free throughout the Sb_2_S_3_ layer thickness, as confirmed by combined studies of energy dispersive X-ray spectroscopy and synchrotron soft X-ray emission spectroscopy, semitransparent Sb_2_S_3_ thin films can be rapidly grown in air by the area-scalable ultrasonic spray pyrolysis method. Integrated into a ITO/TiO_2_/Sb_2_S_3_/P3HT/Au solar cell, a power conversion efficiency (PCE) of 5.5% at air mass 1.5 global (AM1.5G) is achieved, which is a record among spray-deposited Sb_2_S_3_ solar cells. An average visible transparency (AVT) of 26% of the back-contact-less ITO/TiO_2_/Sb_2_S_3_ solar cell stack in the wavelength range of 380–740 nm is attained by tuning the Sb_2_S_3_ absorber thickness to 100 nm. In scale-up from mm^2^ to cm^2^ areas, the Sb_2_S_3_ hybrid solar cells show a decrease in efficiency of only 3.2% for an 88 mm^2^ Sb_2_S_3_ solar cell, which retains 70% relative efficiency after one year of non-encapsulated storage. A cell with a PCE of 3.9% at 1 sun shows a PCE of 7.4% at 0.1 sun, attesting to the applicability of these solar cells for light harvesting under cloud cover.

## Introduction

Modern buildings, especially high-rise buildings, have a large window area available for building-integrated photovoltaics (BIPV). Covering the windows with semitransparent thin film solar cells creates energy-producing solar windows. In addition to current BIPV technology, solar windows could provide advantageous features: they are mountable during construction, they promise an effective utilization of building space, as well as cost and weight savings, and about half of the building electricity demand can be produced on site [1]. Solar windows can be split into two groups: perforated grids of opaque solar cells, such as silicon, or one continuous semitransparent thin film solar cell (dye-sensitized, perovskite, quantum dot, etc.) [1]. Perforated solar windows, comprised of fragments of crystalline Si (c-Si) solar cells, have shown a tendency to overheat and underperform in efficiency (PCE) [2–3]. C-Si grids are also considered visually unappealing for solar windows [4]. Accordingly, thin film solar cells, even with lower PCE, are considered more promising for applications in solar windows [1,4].

The fundamental issue of semitransparent solar cells is a tradeoff between high PCE and high average visible transparency (AVT). The AVT of solar cells must be over 20% to qualify as semitransparent [4]. The PCE and AVT of semitransparent thin film solar cells are listed in the following for reference: dye-sensitized – PCE of 9.2% at 60% AVT [5]; polymer – PCE of 4.0% at 66% AVT [6]; halide perovskite – PCE of 6.4% at 30% AVT [7]. Dye-sensitized, organic, and halide perovskite absorbers are generally sensitive to moisture, especially in combination with sunlight and air [8–10]. At present, tremendous research efforts have been allocated worldwide to increase the long-term stability of these solar cells [11]. As minimizing fabrication cost is crucial for commercialization, solar windows would benefit from a fully inorganic absorber that has superior stability towards moisture and air as well as sunlight.

Sb_2_S_3_ has attractive properties (*E*_g_ ≈ 1.7 eV, absorption coefficient α ≈ 1.8 × 10^5^ cm^−1^ at 450 nm, anisotropic structure, inorganic) as a light absorber for conventional and semitransparent photovoltaic use [12–14]. Sb_2_S_3_ has been incorporated as a solar absorber in photo-electrochemical cells, thin film cells, extremely thin absorber (ETA) cells, and hybrid solar cells based on a planar underlay or on nano- or mesostructured scaffolds [15–22]. Studies on ETA Sb_2_S_3_ cells, which became the basis for respective hybrid solar cells, were pioneered by the teams of Nair, Nezu, and Hodes in the mid-2000s [19,23–24]. The record PCE of 7.5%, achieved with solar cells based on Sb_2_S_3_ grown by chemical bath deposition (CBD) into mesoporous TiO_2_, shows the excellent potential of Sb_2_S_3_ as a PV absorber, and the suitability of its fabrication by chemical methods [20]. Until now, semitransparency aspects of Sb_2_S_3_ solar cells have only been studied by Zimmermann et al., who reported a PCE of 4.25% for a tin-doped indium oxide (ITO)/TiO_2_/Sb_2_S_3_/poly(3-hexylthiophene-2,5-diyl) (P3HT)/Ag solar cell with a 50–70 nm thick Sb_2_S_3_ absorber and a nontransparent 125 nm Ag back contact [21].

TiO_2_ is the most commonly used electron transport material (ETM) in Sb_2_S_3_ solar cells [18,25–32]. SnO_2_ and ZnO have also been employed as the planar ETM, with varying success [33–34]. Conjugated polymers, e.g., P3HT, Spiro-OMeTAD (2,2',7,7'-tetrakis[*N*,*N*-di(4-methoxyphenyl)amino]-9,9'-spirobifluorene), and poly[2,6-(4,4-bis(2-ethylhexyl)-4*H*-cyclopenta[2,1-*b*;3,4-*b*′]dithiophene)-*alt*-4,7-(2,1,3-benzothiadiazole)] (PCPDTBT), are the most popular organic hole transport materials (HTMs) in Sb_2_S_3_ solar cell studies because of the high PCE values [17–18,25,27–31,35]. However, planar cells with inorganic HTMs (which are chemically and thermally more stable and have lower cost), such as CuSCN, NiO*_x_*, and V_2_O_5_, have also shown comparable efficiencies [26,36–37].

As the performance of PV cells highly depends on the quality of the absorber, the development of fabrication techniques to produce high quality Sb_2_S_3_ absorber layers, with few grain boundaries and intra-grain defects is essential to enable commercialization of Sb_2_S_3_-based solar cells [14,38]. The record PCE of 5.77% was achieved with a planar TiO_2_/Sb_2_S_3_/P3HT cell by employing an 87 nm thick Sb_2_S_3_ thin film absorber grown by atomic layer deposition (ALD) [18], whereas a PCE of 4.25% was reported when using Sb_2_S_3_ layers grown by CBD [21]. Unfortunately, the Sb_2_O_3_ impurity phase, which is considered detrimental to PV performance, unavoidably forms in the bulk of the Sb_2_S_3_ thin film when it is grown by CBD from an aqueous solution [18]. In 2018, PCE ≈ 5.7% was achieved for a cell based on a spin-coated Sb_2_S_3_ absorber, and the same group further increased the PCE of this cell to 6.4% by Zn doping during spin-coating of Sb_2_S_3_ [39–40]. Soon after, by doping Sb_2_S_3_ with CsOH, the PCE of planar Sb_2_S_3_ solar cells was boosted from 4.3% to 6.6% [41]. When looking forward to mass production on meter-sized substrates, however, spin-coating cannot be upscaled due to design limitations [42]. Industrialization is feasible only for low-cost, upscalable methods, to the detriment of both conventional ALD and CBD that require several hours to deposit 100 nm thick Sb_2_S_3_ films [18,20–21]. Regarding vacuum deposition methods, a PCE of 3.5% was achieved by thermally evaporating 700 nm of Sb_2_S_3_ onto planar CdS. The main drawbacks of thermal evaporation and conventional ALD as vacuum techniques are the high energy demand and the need for batch processing, which inflates production costs.

As a potent solution-based chemical deposition method, ultrasonic chemical spray pyrolysis (USP) is capable of rapid, area-scalable, roll-to-roll and low-cost in-air deposition of Sb_2_S_3_ layers without imposing limitations on the substrate size [43–44]. A recent paper showed a PCE of 4.6% in solar cells based on Sb_2_(S,Se)_3_ grown onto planar CdS by USP, followed by Se vapor annealing at ≈400 °C. However, pristine Sb_2_S_3_ solar cells consistently yielded a PCE below 0.1% [45]. This clearly illustrates the difficulty of preparing high quality Sb_2_S_3_ absorber layers by USP. A comparative overview of HTMs, deposition methods and PV parameters of solar cells of planar TiO_2_/Sb_2_S_3_/HTM configuration of relevant studies is provided in Table S1 in Supporting Information File 1.

Previously, we observed that the moderate photocurrent density and PCE (of 1.9%) in solar cells based on Sb_2_S_3_ layers grown by USP was due to a discontinuity of the Sb_2_S_3_ layer [28]. In our recent study, we showed that the discontinuity of Sb_2_S_3_ films grown by USP, and likely other chemical methods, is a result of 3D island growth [46]. We demonstrated that by adapting a two-step sequence, whereby amorphous Sb_2_S_3_ layers are first deposited by USP and then crystallized by thermal annealing, compact Sb_2_S_3_ thin films with uniform thickness can be fabricated [46]. Similarly, a two-step procedure to grow compact Sb_2_S_3_ thin films has become common practice for many deposition techniques [18–20,27,29,31,35,47]. To summarize: in order to achieve progress in the various areas of PV applications, e.g., BIPV, and to increase the availability of PV beyond the state-of-the-art in compliance with ever stricter safety and health regulations, novel thin film solar cell designs are required, using abundant non-toxic materials and implementing cost-effective solar cell fabrication technologies.

The aim of this study was to fabricate state-of-the art hybrid solar cells, based on compact thin films of Sb_2_S_3_ deposited by USP in air, by optimizing the thickness of the Sb_2_S_3_ layer, and to consider the influence of cell area, storage time and light intensity on PV performance to investigate their potential for application in semitransparent solar windows. In this study, hybrid solar cells with a maximum PCE of 5.5% at AM1.5G and an AVT of 26% without back contact were fabricated. A PCE of 3.2% was recorded for a solar cell with 88 mm^2^ area. This is the highest PCE in this size category; so far, the PCE of planar Sb_2_S_3_ solar cells has only been reported for ≈1 cm^2^ area (by our group). A solar cell with a PCE of 3.9% at AM1.5G (1 sun) showed a PCE of 7.4% at 0.1 sun, and 10.2% at 0.03 sun, demonstrating the suitability of this solar cell for operation in direct sunlight, as well as under full cloud cover.

## Results and Discussion

### Quality assessment of USP-Sb_2_S_3_ thin films

**Figure 1:**
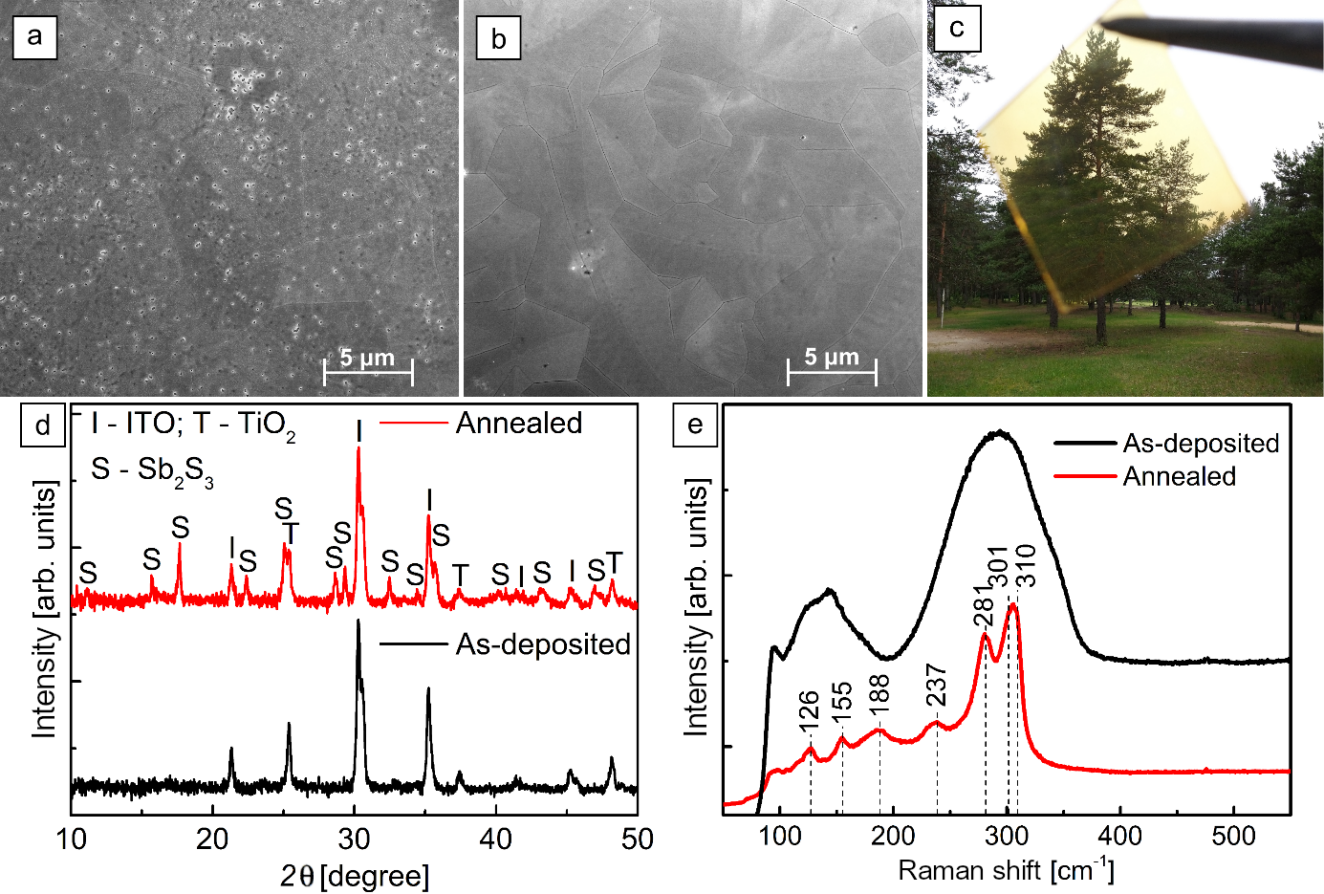
Surface views, by scanning electron microscopy (SEM) of 70 nm (a) and 100 nm (b) thick annealed Sb_2_S_3_ layers on a glass/ITO/TiO_2_ substrate. Photograph (c) of a 5 × 5 cm semitransparent (AVT 26%) stack of glass/ITO/TiO_2_/100 nm annealed Sb_2_S_3_, photographed by J. S. Eensalu. X-ray diffraction patterns (d) and Raman spectra (e) of as-deposited and vacuum-annealed Sb_2_S_3_ layers on glass/ITO/TiO_2_ substrate.

The substrate coverage of Sb_2_S_3_ layers on a glass/ITO/TiO_2_ substrate, annealed in vacuum, depends on the quantity of Sb_2_S_3_ deposited by USP [46]. Thinner (≤70 nm) Sb_2_S_3_ layers contain pin-holes (Figure 1a), whereas thicker (≥100 nm) Sb_2_S_3_ layers fully cover the TiO_2_ ETM (Figure 1b). As the Sb_2_S_3_ film thickness is increased from 70 to 100 nm, the average lateral grains size increases from ≈5 µm (Figure 1a) to ≈10 µm (Figure 1b). An increase in grain size with Sb_2_S_3_ film thickness has been observed by using both physical deposition techniques and chemical deposition techniques [16,32,48]. For reference, the semitransparency of a 5 × 5 cm glass/ITO/TiO_2_/100 nm Sb_2_S_3_ stack, showing an AVT of 26%, is illustrated in a photograph in Figure 1c. The initial results show the excellent perspective of this type of solar cell. However, it should be noted that the AVT requirement for semitransparent solar cells generally refers to the complete stack. Thus, further optimization of the HTM and back contact is needed to attain an AVT in excess of 20% for the complete solar cell.

As-deposited Sb_2_S_3_ layers on glass/ITO/TiO_2_ substrate were amorphous (Figure 1d), as only signals of anatase-TiO_2_ and In_2_O_3_ from the substrate were detected by X-ray diffraction (XRD). In contrast, the XRD pattern of the vacuum-annealed sample matched orthorhombic Sb_2_S_3_ (ICDD PDF 01-075-4012). The Raman spectrum of the as-deposited Sb_2_S_3_ layer contains two broad bands (Figure 1e), which are characteristic of amorphous Sb_2_S_3_ [28,46]. After vacuum annealing, characteristic narrower bands of Sb_2_S_3_ are detected, which is an expected result when crystalline Sb_2_S_3_ is formed [28,46,49]. No traces of additional phases were detected by either XRD or Raman in any glass/ITO/TiO_2_/Sb_2_S_3_ samples. Chlorine, which could originate from the SbCl_3_ precursor, was not detected by energy-dispersive X-ray spectroscopy (EDX) in any sample. Furthermore, the atomic ratio of S to Sb in the annealed Sb_2_S_3_ layers was close to the stoichiometric value of 1.5, as estimated using EDX (Figure S1 in Supporting Information File 1).

**Figure 2:**
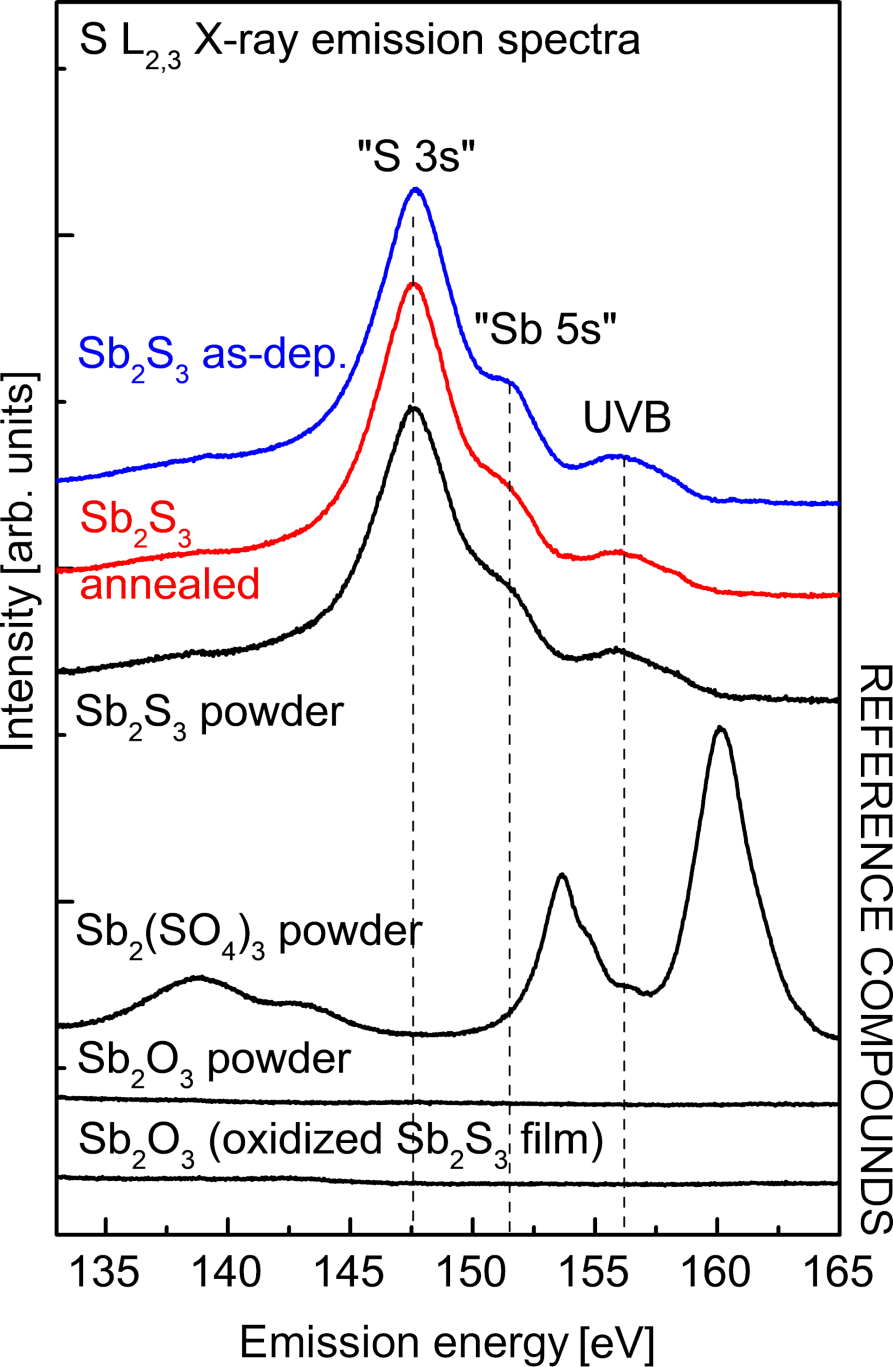
S L_2,3_ XES spectra of two Sb_2_S_3_ films, as-deposited (“as-dep.”, blue) and after post-deposition treatment (“annealed”, red), both on a glass/ITO/TiO_2_ underlay (excitation energy 180 eV). Peaks corresponding to electronic transitions from valence states with strong S 3s and Sb 5s contributions into the S L_2,3_ core holes of the XES initial state, and transitions from the upper valence band (UVB) are indicated. Reference spectra: Sb_2_S_3_ powder, Sb_2_(SO_4_)_3_ powder, Sb_2_O_3_ powder, and an intentionally oxidized Sb_2_S_3_ film. Note the double-peak structure at ≈140 eV in the Sb_2_(SO_4_)_3_ spectrum is ascribed to a 2nd order carbon K emission signal from the support of the powder.

Soft X-ray emission spectroscopy (XES) is an element- and site-specific method that allows for the study of the electronic structure and chemical bonding in materials [50–53]. The attenuation length (*e^−^*^1^) of ≈180 eV soft X-rays in Sb_2_S_3_ is ≈83 nm [54], which makes XES an excellent tool for non-destructively studying the near-surface regions and bulk of thin films [55]. For 50 nm thick Sb_2_S_3_ layers, the XES spectra (Figure 2) probe the chemical states in the entire Sb_2_S_3_ film. The S L_2,3_ XES data in Figure 2 allows three transitions for the Sb_2_S_3_ films and the reference (denoted as “S 3s”, “Sb 5s”, and UVB – upper valence band) to be clearly distinguished. These transitions stem from electronic transitions from valence bands into the S 2p core holes (S L_2,3_) created as the initial state of XES. The transition centered at 147.5 eV is predominantly due to S 3s-derived electronic valence states and appears as the main transition of sulfides [50]. Another peak, as a shoulder for the former, is found at 151 eV and ascribed to Sb 5s-derived states by comparison with band structure and density of states calculations [50]. Lastly, transitions from the upper valence band of Sb_2_S_3_ can be found centered at around 156 eV. These transitions were identified in line with atom-decomposed density of states prediction in the valence band of Sb_2_S_3_, calculated from first principles [56].

The overall spectral shape of the Sb_2_S_3_ thin films, as-prepared and after annealing, agree very well with the Sb_2_S_3_ reference powder. In contrast, no evidence for S–O bonds can be found in the Sb_2_S_3_ thin film spectra, as can be seen by comparing with the reference spectra of Sb_2_(SO_4_)_3_. Sulfate spectra have characteristic line shapes [53]. Thus, the XES study suggests that S is exclusively bonded to Sb in the Sb_2_S_3_ films, throughout its thickness, in the entirety of the analyzed spot size, and both as-deposited and after annealing. The XES study, in addition to the EDX results that showed a S to Sb atomic ratio of 1.5 in the layers, provides further assurance that inclusion of O in the form of a minor impurity phase in the Sb_2_S_3_ layers is likely negligible. Thus, even without further scrutinizing the layer composition, these results already give USP a distinct advantage over aqueous CBD, wherein the inclusion of oxygen is inevitable and traceable [57–59].

To summarize the thin film characterization, we have fabricated polycrystalline, chlorine-free (below EDX detection limit), and oxygen-free (EDX and XES analysis) Sb_2_S_3_ thin films by USP in air. To our knowledge, this is the first report on XES experiments for Sb_2_S_3_ thin films, which, for us, provided the indispensable support of evidence for the claim of the exclusion of oxygen in Sb_2_S_3_ thin films grown by USP in air.

### Development of USP-Sb_2_S_3_ semitransparent solar cells

#### Influence of Sb_2_S_3_ thickness on PV performance of semitransparent Sb_2_S_3_ solar cells

**Figure 3:**
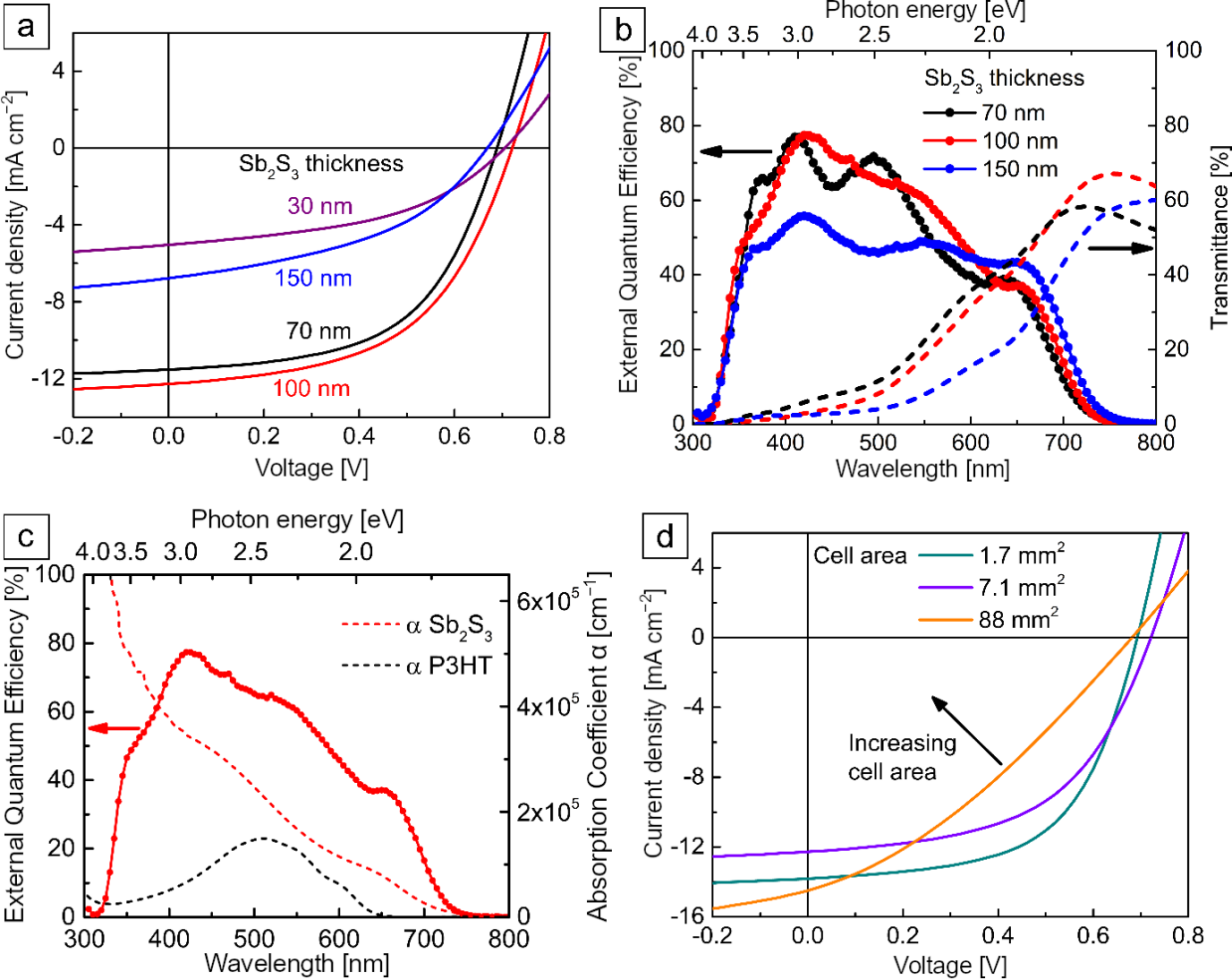
(a) Effect of Sb_2_S_3_ absorber thickness on *J–V* curves at AM1.5G. (b) EQE of solar cells and transmittance of the glass/ITO/TiO_2_/Sb_2_S_3_ stack. (c) EQE of the best-performing solar cell (100 nm Sb_2_S_3_) and absorption coefficients (α) of Sb_2_S_3_ and P3HT. (d) *J–V* curves at AM1.5G of 100 nm Sb_2_S_3_ solar cells of different size.

To investigate the effect of Sb_2_S_3_ film thickness on PV performance of solar cells, we applied 30, 70, 100, and 150 nm thick films of USP-Sb_2_S_3_. By increasing the Sb_2_S_3_ layer thickness from 30 to 100 nm in glass/ITO/TiO_2_/Sb_2_S_3_/P3HT/Au solar cell (Figure 3a), the open-circuit voltage (*V*_OC_) decreased slightly (704 ± 7 mV vs 693 ± 17 mV), the short-circuit current (*J*_SC_) doubled (4.8 ± 0.3 mA cm^−2^ vs 10.3 ± 1.0 mA cm^−2^), the fill factor (FF) increased moderately (43 ± 3% vs 52 ± 3%), and consequently, the PCE increased by a factor of ≈2.5 (1.5 ± 0.1% vs 3.7 ± 0.4%). The highest *V*_OC_ of 726 mV observed in this study is comparable to the highest *V*_OC_ of 732 mV reported for planar TiO_2_/Sb_2_S_3_ solar cells, where Sb_2_S_3_ was grown by chemical bath deposition [21]. Increasing the Sb_2_S_3_ layer thickness further to 150 nm causes all photoconversion parameters to plummet; an expected result in the case of increased recombination losses in the bulk of the Sb_2_S_3_ absorber layer [60]. The FF is adversely affected by large values of series resistance (*R*_S_) and by small values of shunt resistance (*R*_SH_) [61]. The cells with 70 nm thick Sb_2_S_3_ had the highest FF of 57 ± 4%, incidentally coinciding with the smallest of *R*_S_ and the largest of *R*_SH_, whereas the FF was slightly smaller in cells with 100 nm thickness, mostly due to a smaller *R*_SH_. Compared to cells with 70–100 nm thick Sb_2_S_3_ layers, the FF was smaller by around 10% in cells with both the thinnest (30 nm) and the thickest (150 nm) Sb_2_S_3_ layers. The decrease in the FF in these cells could mainly be attributed to the occurrence of *R*_S_ over 10 Ω cm^2^ (Table 1). In particular, *R*_S_ could be inflated in cells with thin (30 nm) Sb_2_S_3_ layers, because the mobility of charge carriers is likely impeded by numerous grain boundaries owing to the smaller grain size in thinner films. On the other end, *R*_S_ is also over 10 Ω cm^2^ in the cells with overly thick Sb_2_S_3_ layers (150 nm), which is ascribed to the ohmic resistance of the thicker absorber. In this study, the resistivity (ρ) of 100–150 nm thick Sb_2_S_3_ films on glass/TiO_2_ substrate was measured by the collinear four-wire technique and by van der Pauw measurements to be in the range of 2–3 × 10^6^ Ω cm, as anticipated.

**Table 1:** Photoconversion parameters^a^ of solar cells as a function of Sb_2_S_3_ film thickness. The best results are given in parentheses.

Sb_2_S_3_ [nm]	*V*_OC_ [mV]	*J*_SC,_*_I_*_–_*_V_* [mA cm^−2^]	*J*_SC,EQE_	FF [%]	PCE [%]	*R*_S_ [Ω cm^2^]	*R*_SH_ [kΩ cm^2^]	Nr^b^

30	704 ± 7^c^ (705)	4.8 ± 0.3 (5.00)	– (6.78)	43 ± 3 (46)	1.5 ± 0.1 (1.6)	14 ± 2.1 (18)	0.5 ± 0.1 (0.6)	8
70	670 ± 8 (691)	7.5 ± 0.6 (11.5)	– (10.3)	57 ± 4 (55)	2.9 ± 0.2 (4.4)	7.4 ± 0.6 (6.0)	2.2 ± 1.0 (1.1)	9
100	693 ± 17 (726)	10.3 ± 1.0 (12.3)	– (10.9)	52 ± 3 (52)	3.7 ± 0.4 (4.7)	7.6 ± 1.5 (5.9)	0.9 ± 0.3 (0.7)	36
150	638 ± 16 (669)	4.3 ± 1.1 (6.88)	– (9.60)	44 ± 1 (43)	1.2 ± 0.3 (2.0)	26 ± 5.2 (19)	0.7 ± 0.2 (0.4)	8

^a^Measurement conditions: 100 mW cm^−2^, AM1.5G, cell active area 7.1 mm^2^; ^b^Number of measured cells; ^c^Standard deviation.

Figure 3b shows the external quantum efficiency (EQE) of solar cells with 70, 100 and 150 nm thick Sb_2_S_3_ thin films and transmittance of solar cells without P3HT/Au back contact. The AVT of the stacks of glass/ITO/TiO_2_/Sb_2_S_3_ with 70, 100, and 150 nm of Sb_2_S_3_ is 28%, 26%, and 16%, respectively in the 380–740 nm wavelength range (Figure 3b). Thus, the 150 nm thick Sb_2_S_3_ film is already too opaque for it to qualify as a semitransparent absorber layer. According to the EQE, appreciable photoelectric conversion in these cells occurs in the 320–750 nm wavelength range. The observed EQE onset at 750 nm corresponds to a band gap of 1.65 eV of crystalline Sb_2_S_3_. Cells with 70 and 100 nm thick Sb_2_S_3_ film showed the best EQE values, reaching almost 80% EQE at around 425 nm wavelength, which is almost the maximum realistically attainable EQE. The decreased EQE at higher wavelengths is common for solar cells with a chemically deposited Sb_2_S_3_ absorber [18,21]. The average and best photoconversion parameters calculated from the *J–V* curves and EQE are presented in Table 1. Compared to *J*–*V*-derived PV parameters of planar TiO_2_/Sb_2_S_3_/HTM solar cells (Supporting Information File 1, Table S1), the PCE achieved in this study for a 1.7 mm^2^ cell area (5.5%) and 7.1 mm^2^ cell area (4.7%) is among the top values achieved in the last five years, and close to the record PCE of planar solar cells based on pristine Sb_2_S_3_. The mismatch in *J*_SC_ calculated from *J–V* and EQE likely stems from the difference in light intensity during *J–V* and EQE measurements, coupled with a strong dependence of photoelectric conversion efficiency on light intensity in these solar cells, as will be discussed later on. The EQE shoulder at around 650 nm (Figure 3b), indicates the presence of a beneficial phenomenon called the optical spacer effect, which can occur in solar cells with a very thin absorber [21,62–63]. The optical spacer effect increases the EQE at above 650 nm, where P3HT does not absorb light. The magnitude of the gain in EQE due to this effect depends on the thickness of the HTM and that of the absorber [21]. The optical spacer effect can have a strong influence on the EQE when the thickness of the absorber is around 100 nm or less [62]. Otherwise, most of the incident light is absorbed before reaching the optical spacer layer and the optical spacer effect is not seen. The optical spacer effect is illustrated in the EQE spectrum (Figure 3c) of one of the best-performing devices (100 nm Sb_2_S_3_, 7.1 mm^2^) coupled with the absorption coefficient curves of Sb_2_S_3_ and P3HT. The transmittance of light to the absorber is limited at higher photon energies by the onset of absorption of TiO_2_ at 3.0 eV and ITO at 3.6 eV. The P3HT layer, however, does not contribute to the generation of photocurrent [14,21]. On the contrary, any photogeneration within the P3HT is known to have an adverse effect on *J*_SC_ and FF [14,21]. Lastly, the EQE of cells with 150 nm thick Sb_2_S_3_ indicates a decline of the collection of photogenerated carriers in the 350–600 nm wavelength range (Figure 3b). The decrease is more drastic at lower wavelengths, which are more rapidly dampened in Sb_2_S_3_, as evident from the absorption coefficient (Figure 3c), and related photoexcitation in the Sb_2_S_3_ occurs closer to the side of incidence, i.e., the ETM/Sb_2_S_3_ interface. Hence, we are led to conclude that the holes photogenerated near the ETM/Sb_2_S_3_ interface, which must travel the farthest towards the HTM, face mobility issues when traversing the thickest (150 nm) absorber layer. On the other end, at wavelengths above 600 nm, the benefit of using layers thicker than 100 nm to absorb more light is clearly seen through increased EQE, as expected. For reference, the penetration depth for light of 600 nm wavelength is about 100 nm, assuming α = 1 × 10^5^ cm^−1^ (Figure 3c). Electron mobility tends to be greater in semiconductors when compared to hole mobility, although the efficacy of electron transport is also subject to change when the absorber thickness is varied. In this particular case, however, the spacer effect also occurs in the EQE spectra at wavelengths above 600 nm, for which the light reaches deepest into Sb_2_S_3_ and closer to the back electrode. Hence, more sophisticated analyses might be appropriate for the complete depiction of the impact of Sb_2_S_3_ thickness on electron transport. The existence of the optical spacer effect can also have a negative impact. Even some tens of nanometers off of the optimum HTM thickness at constant absorber layer thickness could drastically decrease the *J*_SC_; thus it is crucial to ensure uniform thickness of P3HT throughout the whole area of the solar cell [62].

#### Influence of cell area on PV performance of semitransparent Sb_2_S_3_ solar cells

**Figure 4:**
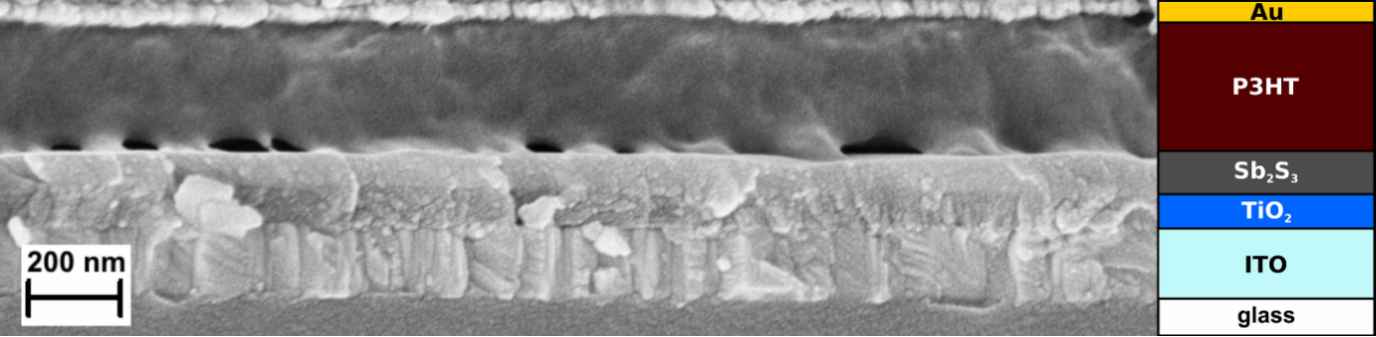
SEM cross-section of the best-performing 5.5% PCE solar cell (100 nm Sb_2_S_3_) and the corresponding device schematic.

**Figure 5:**
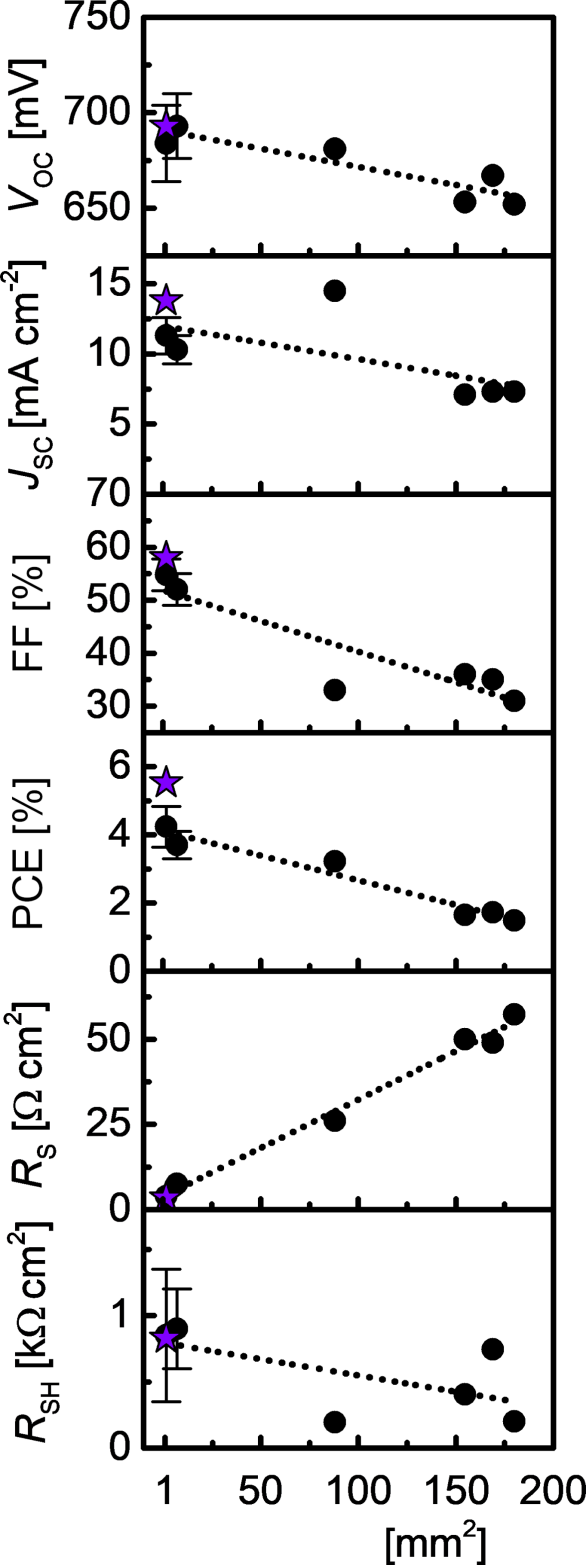
Photoconversion parameters of 100 nm Sb_2_S_3_ solar cells under illumination at AM1.5G as a function of cell area. The dotted lines are a guide to the eye; the purple star signifies the best-performing 1.7 mm^2^ cell with 5.5% PCE. The vertical bars signify standard deviation, and black dots represent either average values of smaller cells (area <10 mm^2^), or individual values of larger cells (area >10 mm^2^).

To investigate the effect of enlarging cell area on PV performance, we fabricated cells with active area ranging from 1.7 to 180 mm^2^ and calculated the photoconversion parameters from *I–V* curves measured at 100 mW cm^−2^ with AM1.5G (Figure 3d). The cross-sectional SEM view of the best solar cell with 100 nm of Sb_2_S_3_ is presented in Figure 4 alongside the corresponding device schematic. As the cell area was increased from 1.7 to 180 mm^2^, *V*_OC_, *J*_SC_, FF, and *R*_SH_ all decreased linearly at different rates, but at the same time, *R*_S_ increased substantially (Figure 5). As a result, the PCE decreased from 4.2 ± 0.6% to 1.6%. The highest PCE of 5.5%, 4.7%, and 3.2% at AM1.5G (Table 2) was obtained in the three best-performing cells with 1.7 mm^2^, 7.1 mm^2^ and 88 mm^2^ area, respectively. The statistical variance of photoconversion parameters (*V*_OC_, *J*_SC_, FF, PCE, *R*_S_, *R*_SH_) of 36 cells of 7.1 mm^2^ active area with 100 nm Sb_2_S_3_ is presented in Figure S2 in Supporting Information File 1.

**Table 2:** Photoconversion parameters^a^ of solar cells as a function of active area. The best results are given in parentheses.

Area [mm^2^]	*V*_OC_ [mV]	*J*_SC,_*_I_*_–_*_V_* [mA cm^−2^]	FF [%]	PCE [%]	*R*_S_ [Ω cm^2^]	*R*_SH_ [kΩ cm^2^]	Nr^b^

1.7	684 ± 20^c^ (693)	11.3 ± 1.3 (13.8)	55 ± 3 (58)	4.3 ± 0.6 (5.53)	3.9 ± 1.2 (3.3)	0.8 ± 0.5 (0.8)	37
7.1	693 ± 17 (726)	10.3 ± 1.0 (12.3)	52 ± 3 (52)	3.7 ± 0.4 (4.67)	7.6 ± 1.5 (5.9)	0.9 ± 0.3 (0.7)	36
88	681	14.5	33	3.22	26	0.2	1
155	653	7.1	36	1.65	50	0.4	1
169	667	7.3	35	1.72	49	0.8	1
180	652	7.3	31	1.49	57	0.2	1

^a^Measurement conditions: 100 mW cm^−2^, AM1.5G, Sb_2_S_3_ thickness 100 nm; ^b^Number of measured cells; ^c^Standard deviation.

Despite commendable *V*_OC_ (682 mV) and *J*_SC_ (14.5 mA cm^−2^), the FF is substantially smaller (33%) in larger (≈100 mm^2^) cells when compared to <10 mm^2^ cells (Table 2) due to about three times larger *R*_S_ (26 Ω mm^2^) and about three times smaller *R*_SH_ (190 Ω mm^2^) under both illuminated (Figure 3d) and dark conditions (Figure S3 in the Supporting Information File 1). Also, the photocurrent loss in larger cells originates from the increase in *R*_S_ alongside the decrease in *R*_SH_ (Table 2). In addition, the probability of a given cell to exhibit photocurrent loss, and a resulting decrease in FF, increases proportionally with area due to unforeseen thickness fluctuations and a resultant mismatch in the thicknesses of TiO_2_, Sb_2_S_3_, and P3HT layers, assuming to be primarily due to a uniform distribution of defects. Upon scribing large cells (>100 mm^2^) into several smaller ≈0.1 mm^2^ cells, all photoconversion parameters of the cells with USP-grown Sb_2_S_3_ ended up showing values like those of individual cells with similar sizes, as has previously been demonstrated for structured ETA-Sb_2_S_3_ cells [14]. In a study of SnO:F/CdS/Sb_2_(S,Se)_3_/C/Ag solar cells of 20–80 mm^2^ area, a similar trend of lower PCE in larger cells was described (from 6.2% at 20 mm^2^ to 5.7% at 60 mm^2^) [64]. We perceive the most concerning issues with up-scaling of planar solar cells with Sb_2_S_3_ grown by USP as the following: (1) enlarging the cell area causes FF loss, possibly because of minute, nontrivial discrepancies in layer thickness of Sb_2_S_3_, and particularly P3HT; (2) enlarging the cell area introduces loss in *J*_SC_ and loss in FF due to the large resistivity of the absorber layer. It appears that the PCE on the level of small cells (<10 mm^2^) can only be achieved in larger cells (>100 mm^2^) if the TiO_2_, Sb_2_S_3_ and P3HT layers are uniform to the precision of a few nanometers in thickness [14,21,34,62]. Therefore, device performance is not only highly dependent on the deposition technique for its ability to produce pure phase Sb_2_S_3_, but also on the capability of the specific deposition equipment to produce films with superior uniformity in thickness, i.e., nanometer precision, which USP can provide after some optimization.

#### Influence of storage time on PV performance of semitransparent Sb_2_S_3_ solar cells

**Figure 6:**
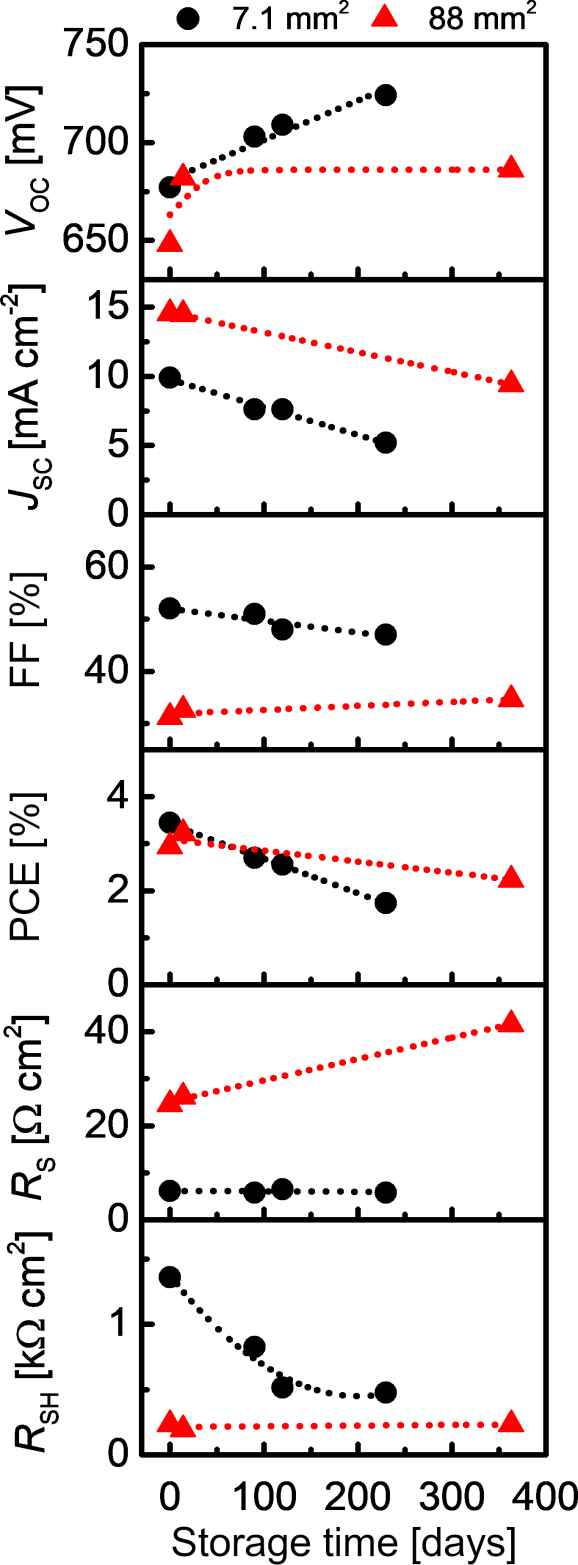
Photoconversion parameters of (≈100 nm Sb_2_S_3_) solar cells at AM1.5G as a function of storage time. The dotted lines are a guide to the eye. Black circles refer to 7.1 mm^2^ cell area, and red triangles refer to 88 mm^2^ cell area. Samples composed of several small cells with P3HT only partially covered by Au were kept without any particular artificial conditions or encapsulation, and between measurements, were exposed to air and cycles of both indoor fluorescent tube irradiation and daylight for up to 363 days at ≈20 °C, RH <30%.

The stability of solar cells is paramount to ensure long-term performance under operation conditions, in turn maximizing return on investment. We investigated the stability of photoconversion parameters of cells of 7.1 mm^2^ and 88 mm^2^ area with USP-grown Sb_2_S_3_ by keeping the cells at rest in *V*_OC_ condition for 230 days at room temperature (RT), relative humidity (RH) <30%, and exposed to both indoor light and daylight incident through the laboratory windows (Figure 6). In the 7.1 mm^2^ cell, over 230 days, *V*_OC_ increased slightly, whereas *J*_SC_ was halved, FF decreased slightly due to three times smaller *R*_SH_, and, as a result PCE was halved in the tested 7.1 mm^2^ cell (Figure 5, numeric data in Table S2 in the Supporting Information File 1), whereas *R*_S_ remained constant. In comparison, a trend of PCE decreasing from 3.7% to 1.5% after 300 days of aging was observed in ETA cells with a TiO_2_/Sb_2_S_3_/CuSCN structure [24]. The *V*_OC_ of the 88 mm^2^ cell increased from 648 mV to 682 mV after 14 days (Figure 5), and remained constant after 363 days of storage, unlike the linear increase of 20 mV per 100 days observed in the 7.1 mm^2^ cell. The reason for this discrepancy is still under question and requires further study. A partially reversible increase of *V*_OC_ over time due to humidity in air is common for solar cells containing organic materials [9]. *J*_SC_ declines linearly at a similar rate in both cells, 2.0 mA cm^−2^ per 100 days for the small cell, and 1.4 mA cm^−2^ per 100 days for the 88 mm^2^ cell, independent of the initial *J*_SC_ value. The linear decrease of *J*_SC_ during aging in light or dark conditions correlates with the general trend in organic PV [9], meaning the stability of Sb_2_S_3_ hybrid solar cell hinges on the stability of the chosen HTM. The FF increased slightly in the 88 mm^2^ cell, opposite to the slight decrease in the smaller cell. Consequently, the PCE of the larger cell decreases by ≈0.2% per 100 days, that is at a slower rate compared to the decrease of 0.7% per 100 days for the small cell. According to Hintz et al., the in-flux of moisture and oxygen from air presumably leads to degradation of P3HT [65]. We suppose that this process occurs more slowly in the larger cell, which could explain why the larger cell retains more PCE over time compared to the smaller cell. Another discrepancy appears when examining the *R*_S_ and *R*_SH_. The *R*_S_ of the large cell increases linearly by 4.5 Ω cm^2^ per 100 days, whereas *R*_S_ is constant for the small cell. The opposite occurs for *R*_SH_, where the small cell rapidly loses *R*_SH_ over the first 120 days, but the initial *R*_SH_ in the large cell is retained after one year. The decrease in PCE over time observed in both 7.1 mm^2^ and 88 mm^2^ area solar cells is probably due to moisture-assisted oxidation of P3HT [65]. Thus, to inhibit loss of PCE over time, encapsulation of the solar cell from moisture and oxygen is advised. Deng et al. studied the effect of storage time on the PCE of ITO/TiO_2_/as-deposited Sb_2_S_3_/P3HT/Au, and ITO/TiO_2_/Sb_2_S_3_ (Se-annealed)/Au solar cells under continuous illumination by measuring *J*–*V* every 24 h [66]. The Se-annealed sample experienced a net gain in PCE in the first 24 h, which was retained over 400 hours of illumination [66]. The sample containing P3HT lost all PCE after 150 hours of illumination, mainly because of the loss of *J*_SC_ [66]. In the same study, it was shown that solar cells without the P3HT layer, with both as-deposited and Se-annealed Sb_2_S_3_ absorber, did not experience a quantifiable loss of PCE over six months of storage in air [66]. Assuming that the materials properties of the layers in these solar cells are similar to the corresponding layers in this study, we argue that the loss of PCE after storage in air is probably caused by the organic P3HT layer. In conclusion, we have demonstrated that increasing the active area of the cell helps to retain PCE of the solar cell based on USP-grown Sb_2_S_3_ absorber over extended periods of time, and this dependence on cell area certainly warrants more thorough investigation.

#### Influence of light intensity on PV performance of semitransparent Sb_2_S_3_ solar cells

**Figure 7:**
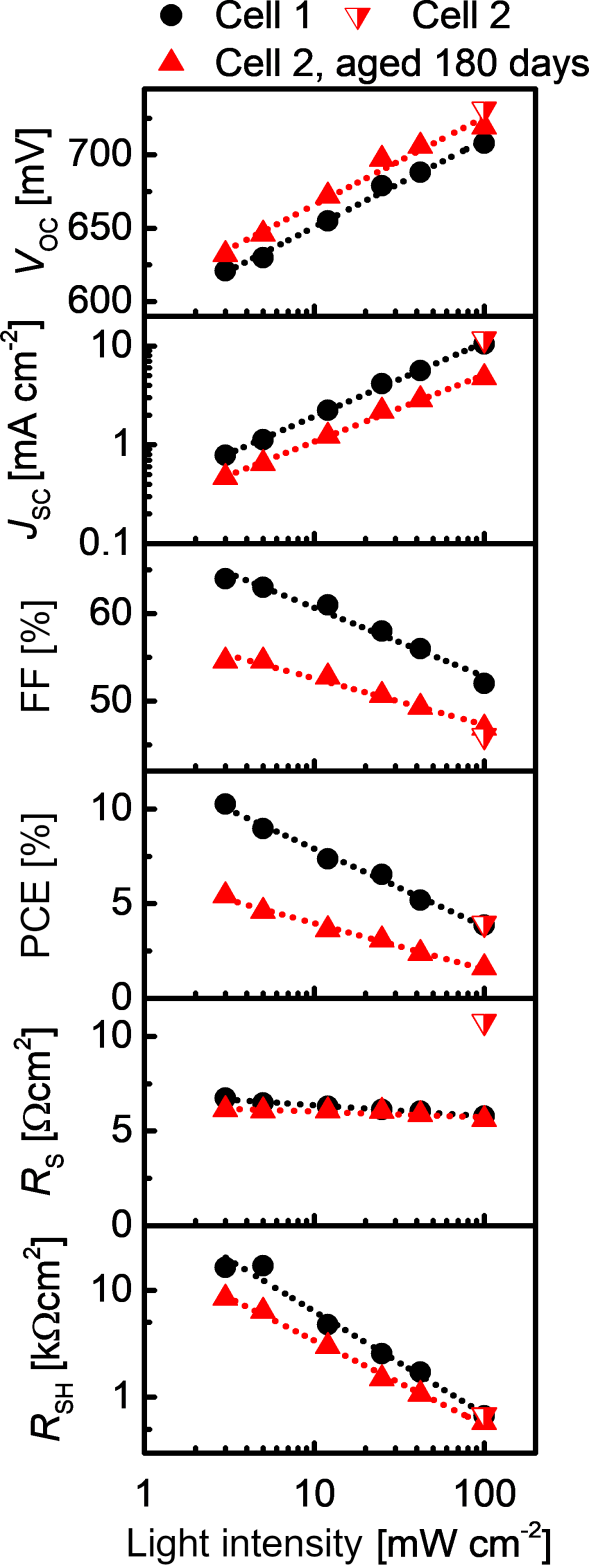
Photoconversion parameters of 100 nm Sb_2_S_3_ solar cells without aging (black dots) or after 180 days of aging (red triangles) as a function of light intensity. The dotted lines are a guide to the eye. The light intensity was attenuated from AM1.5G, 100 mW cm^−2^ with the use of metal mesh gray filters.

The intensity of incident light in real working conditions of solar cells is not constant and is rarely at standard brightness, requiring solar cells to perform well at standard light intensity as well as at attenuated light intensities. In addition, close inspection of the dependence of photoconversion parameters on light intensity could provide valuable information about the cause of *J*_SC_ and FF losses in the solar cell [47]. We investigated the *I*–*V* output of cells with a USP-grown Sb_2_S_3_ absorber at a number of different illumination intensities between 3 and 100 mW cm^−2^. A constant device temperature was maintained to avoid introduction of additional uncertainty to the measurements. The light intensity was attenuated by using metal mesh gray filters. By decreasing the incident light intensity from 100 to 3 mW cm^−2^, *V*_OC_ and *J*_SC_ decreased, as expected, whereas *R*_SH_, *R*_S_ and FF increased (Figure 7). Overall, the PCE increased from 3.9% at 100 mW cm^−2^, AM1.5G, to over 10% at 3 mW cm^−2^. The increase in PCE when lowering light intensity is hereby taken as characteristic of Sb_2_S_3_-based solar cells [19,47]. Curiously, the tendency of change in PCE for Sb_2_S_3_ solar cells is opposite to that of monocrystalline Si solar cells at lower light intensity [67]. In comparison, after aging a solar cell under ambient light and RT for 180 days, in the same conditions as in the storage time test (Figure 5), the PV parameters follow similar trends depending on light intensity (Figure 6). The elevated PCE in freshly prepared and aged cells with USP-grown Sb_2_S_3_ is related to an associable gain of FF and *R*_SH_ at decreased light intensity. The exact reasons for this dependence are yet to be clarified. An investigation on the low light intensity behavior of Cu- and Se-doped Sb_2_S_3_-based hybrid solar cells showed that the PCE of these cells was also significantly higher at lower light intensity (2.12% at 25 mW cm^−2^ and 9.03% at 5 mW cm^−2^), and it was concluded that the behavior was similar to amorphous Si solar cells [68]. In the case of amorphous Si solar cells, the decrease in FF at increasing light intensity was connected to the increasing electric field inside the solar cell [69]. Essentially, if the quality of the solar cell is improved, the PCE at higher light intensity will increase and approach the PCE at low light intensity [69]. While the explanations for many phenomena in this structure are still under discussion, inorganic ETM/Sb_2_S_3_/organic HTM solar cells demonstrate excellent applicability for 1–10 mW cm^−2^ light harvesting, a commonly observed range of light intensity when partial or full cloud cover is present. The durability of these solar cells is expected to be a manageable concern under attenuated light.

## Conclusion

Semitransparent and oxide-free thin films of crystalline Sb_2_S_3_ were fabricated at low temperature using two steps, an initial non-vacuum growth by facile spray pyrolysis (USP) at 200 °C, followed by a low-temperature annealing in a non-oxygen containing environment at 170 °C. This process is compatible with existing window glass manufacturing technology. By integrating semitransparent thin films of Sb_2_S_3_ with optimized thickness of 100 nm in the planar ITO/TiO_2_/Sb_2_S_3_/P3HT/Au hybrid solar cell, a PCE of 5.5% and an AVT of 26% in ITO/TiO_2_/Sb_2_S_3_ were achieved. The PCE and AVT combinations obtained in this study are similar to those reported for other semitransparent thin film solar cell configurations. The feasibility of fabricating large-area lab-scale Sb_2_S_3_ solar cells by the USP method is demonstrated by achieving a PCE of 3.2% at 88 mm^2^ solar cell area, laying the foundation for further improvements in scalability. Furthermore, over the span of a year, the 88 mm^2^ non-encapsulated solar cell stored under standard office conditions showed half the rate of aging and an increased stability towards humidity and air when compared to smaller cells (<10 mm^2^ area). We consider the trend of increased PCE at decreased light intensity observed in USP-Sb_2_S_3_ solar cells favorable for light conversion under cloud cover. It is clear that the key to further increase the efficiency, area-scalability, and durability of opaque and semitransparent Sb_2_S_3_ solar cells lies in tuning of the layers and interfaces of the ETM/Sb_2_S_3_/HTM stack. Considering the potential benefits of the capability of USP for large-scale production, extensive cost-savings could be achieved by depositing all component layers in the Sb_2_S_3_-solar cell by ultrasonic spray pyrolysis, further accentuating facile integration in solar window glass production.

## Experimental

### Solar cell fabrication

All chemicals were sourced from Sigma-Aldrich and used as-purchased without further processing. ITO covered (25 Ω sq^−1^, ZSW) soda-lime glass substrates were cleaned with deionized water, ethanol, deionized water, H_2_SO_4_ (1% w/w), and rinsed with deionized water before drying at 105 °C in air. Then, a dense, compact TiO_2_ layer was grown on glass/ITO by ultrasonic spray pyrolysis in air from 0.1 M titanium tetraisopropoxide (98% v/v) and 0.4 M acetylacetone (99% v/v) dissolved in ethanol (96.6% v/v) according to a previously published procedure [28,46,70]. After deposition, the glass/ITO/TiO_2_ stack was annealed at 450 °C for 30 min in air to form anatase.

Amorphous layers of Sb_2_S_3_ were deposited by ultrasonic spray pyrolysis in air from a solution of SbCl_3_ (99% w/w) and SC(NH_2_)_2_ (98% w/w), Sb/S molar ratio 1:3, dissolved in methanol (99.8% v/v), according to a previously published procedure [46]. The Sb_2_S_3_ thin film thickness was controlled by varying the concentration of SbCl_3_ and SC(NH_2_)_2_ (1:3) in methanol. The Sb_2_S_3_ thin films were crystallized by annealing in vacuum (≤4 × 10^−6^ torr) at 170 °C for 5 min.

We observed that without annealing of Sb_2_S_3_, the solar cells of the same structure showed a *V*_OC_ of about 700 mV, but virtually no *J*_SC_, producing PCE <0.1%. All solar cells henceforth were based on annealed Sb_2_S_3_ thin films.

P3HT, as the hole transport material (HTM), was applied by immersing samples into a room-temperature solution of regioregular P3HT (2% w/w) dissolved in chlorobenzene (99.5% v/v), then dried at 50 °C for 10 min in air, and further dried in vacuum (≤4 × 10^−6^ torr) for 5 min. The solar cells were completed by depositing the Au counter electrode by thermal evaporation in vacuum (<2 × 10^−4^ torr). The cells with areas of 1.7 mm^2^ and 7.1 mm^2^ were prepared with the use of perforated metal masks. The area of cells larger than 7.1 mm^2^ was defined by mechanically scribing contacts after Au deposition.

### Thin film characterization

The characterization methods employed in this study, except for XRD and XES, have already been described in detail elsewhere [28]. The structure and phase composition were characterized by XRD (Rigaku Ultima IV, θ-2θ, Cu Kα_1_ λ = 1.5406 Å, 40 kV, 40 mA, step 0.02°, 5° min^−1^, Si strip detector D/teX Ultra) and Raman spectroscopy (Horiba Labram HR 800, backscattering mode, ≈143 µW µm^−2^). The elemental composition of glass/ITO/TiO_2_/Sb_2_S_3_ samples and solar cell cross sections were recorded in the combined energy dispersive X-ray spectrometer (Bruker spectrometer, ESPRIT 1.8, 7 kV) and scanning electron microscope (Zeiss HR FESEM Ultra 55, 4 kV) system. The surface morphology of glass/ITO/TiO_2_/Sb_2_S_3_ samples was recorded in a HR-SEM (Helios NanoLab 600, FEI Company). The optical properties were measured using a UV–vis–NIR spectrophotometer (Jasco V-670, integrating sphere, air reference). The AVT was calculated as the arithmetic average of total transmittance of the glass/ITO/TiO_2_/Sb_2_S_3_ stack in the 380–740 nm wavelength range by using Equation 1 [71]:

[1] $$\text{AVT}\left(\%\right)=\frac{\int_{380}^{740}T\left(\lambda \right)\text{d}\left(\lambda \right)}{740-380},$$

where λ is the wavelength, and *T*(λ) (%) is the total transmittance at λ. The resistivity of Sb_2_S_3_ layers on glass/TiO_2_ substrate was measured at room temperature in dark by using the van der Pauw technique (MMR Technologies H50) and collinear four-wire *I–V* sensing (Eco Chemie BV, AutoLab PGSTAT302). The contact material for both measurements was deposited from an aqueous graphite ink from Alfa Aesar. S L_2,3_ soft X-ray emission spectra of Sb_2_S_3_ were measured using the SALSA endstation [72], at the open port of Beamline 8.0.1 of the Advanced Light Source (ALS), at Lawrence Berkeley National Laboratory (LBNL). The Sb_2_S_3_ films were excited with a photon energy of 180 eV, and the emitted X-rays at the S L_2,3_ edge were recorded as a function of energy. The reference chemicals for XES measurements were purchased from Alfa Aesar (Sb_2_S_3_ and Sb_2_O_3_ powders, both 99.999% w/w) and Sb_2_(SO_4_)_3_ powder (99.91% w/w) from Chemsavers.

### Solar cell characterization

The current–voltage (*I–V*) curves of the solar cells were measured by using a factory-calibrated solar simulator (Newport Oriel Sol3A class AAA) that provided AM1.5G, 100 mA cm^−2^ light intensity, a metal mask with adjustable aperture area, and a source meter. The light intensity was regulated for the light intensity dependence measurements using gray filters (metal meshes with varied hole size). The external quantum efficiency (EQE) spectra were measured using a monochromatized light source (Newport 300 W Xenon lamp, 69911 with a monochromator Newport Cornerstone 260), a digital lock-in detector (Merlin) and a factory-calibrated Si reference detector. The integrated short-circuit current density (*J*_SC_) from EQE was calculated in AM1.5G conditions with the online tool Open Photovoltaics Analysis Platform and compared with the *J*_SC_ obtained from the *I–V* measurements.

## Supporting Information

Additional literature data of Sb_2_S_3_ solar cells, EDX data, statistical data of PV parameters of the optimized solar cell, dark *J*–*V* scans, and numeric data of the solar cell aging experiment.

File 1 Additional data.
