# Supplementary material for: Semitransparent Sb2S3 thin film solar cells by ultrasonic spray pyrolysis for use in solar windows
Source: Beilstein J Nanotechnol. 2019 Dec 6;10:2396–409. doi: 10.3762/bjnano.10.230 (PMC6902894; doi:10.3762/bjnano.10.230)
Supplement: File 1 — Additional data. [file Beilstein_J_Nanotechnol-10-2396-s001.pdf]

BEILSTEIN JOURNAL OF NANOTECHNOLOGY

Supporting Information

for

Semitransparent Sb_2S_3 thin film solar cells by ultrasonic spray pyrolysis for use in solar windows

Jako S. Eensalu, Atanas Katerski, Erki Kärber, Lothar Weinhardt, Monika Blum, Clemens Heske, Wanli Yang, Ilona Oja Acik and Malle Krunks

Beilstein J. Nanotechnol. **2019,** *10,* 2396–2409. doi:10.3762/bjnano.10.230

Additional data

License and Terms: This is a supporting information file under the terms of the Creative Commons Attribution License (http://creativecommons.org/licenses/by/4.0). Please note that the reuse, redistribution and reproduction in particular requires that the authors and source are credited.
The license is subject to the *Beilstein Journal of Nanotechnology* terms and conditions: (https://www.beilstein-journals.org/bjnano)

Table S1: Overview of Sb_2S_3 deposition methods, hole transport materials (HTM), and photoconversion parameters of the most efficient solar cells in planar TiO_2/Sb_2S_3/HTM configuration, as found in literature. Reported measurement conditions: 100 mW cm^{-2}, AM 1.5G.

Sb_2S_3 deposition method	HTM	V_{OC} mV	J_{SC} mA cm^{-2}	FF %	PCE %	Area cm^2	Year	Ref.
USP	P3HT	693	13.8	58	5.5	0.017	2019	This study
USP	P3HT	726	12.3	52	4.7	0.071	2019	This study
USP	P3HT	682	14.5	33	3.2	0.88	2019	This study
Spin-coat.	Spiro-OMeTAD	690	17.3	55	6.6[a]	0.12	2019	[1]
Spin-coat.	Spiro-OMeTAD	630	13.3	51	4.3	0.12	2019	[1]
Spin-coat.	Spiro-OMeTAD	660	14.3	60	5.7	0.12	2018	[2]
Spin-coat.	V_2O_5	590	15.3	53	4.8	0.12	2018	[3]
Spin-coat.	NiO_x	590	14.5	41	3.5	0.12	2018	[4]
Spin-coat.	Spiro-OMeTAD	647	17.2	57	6.4[b]	0.12	2018	[5]
Spin-coat	Spiro-OMeTAD	654	14.6	58	5.5	0.12	2018	[5]
Spin-coat.	P3HT	611	15.2	52	4.8	0.16	2018	[6]
Thermal Evapor.	Spiro-OMeTAD	620	10.7	56	3.8	0.10	2018	[7]
CBD	P3HT	550	10	57	3.2	0.090	2018	[8]
Ink-print	P3HT	520	7.8	40	1.5	0.13	2017	[9]
USP	P3HT	618	6.0	51	1.9	0.017	2016	[10]
USP	P3HT	635	5.0	42	1.3	1.0	2016	[10]
CBD	P3HT	732	9.3	62	4.3	0.13	2015	[11]
ALD	P3HT & PEDOT:PSS[c]	667	14.9	58	5.8	0.16	2014	[12]
CBD	P3HT & PEDOT:PSS	489	9.6	46	2.2	0.16	2014	[12]

[a]Cesium-doped

[b]Zinc-doped

[c]poly(3-4-ethylenedioxythiophene) doped with poly(4-styrenesulfonate)

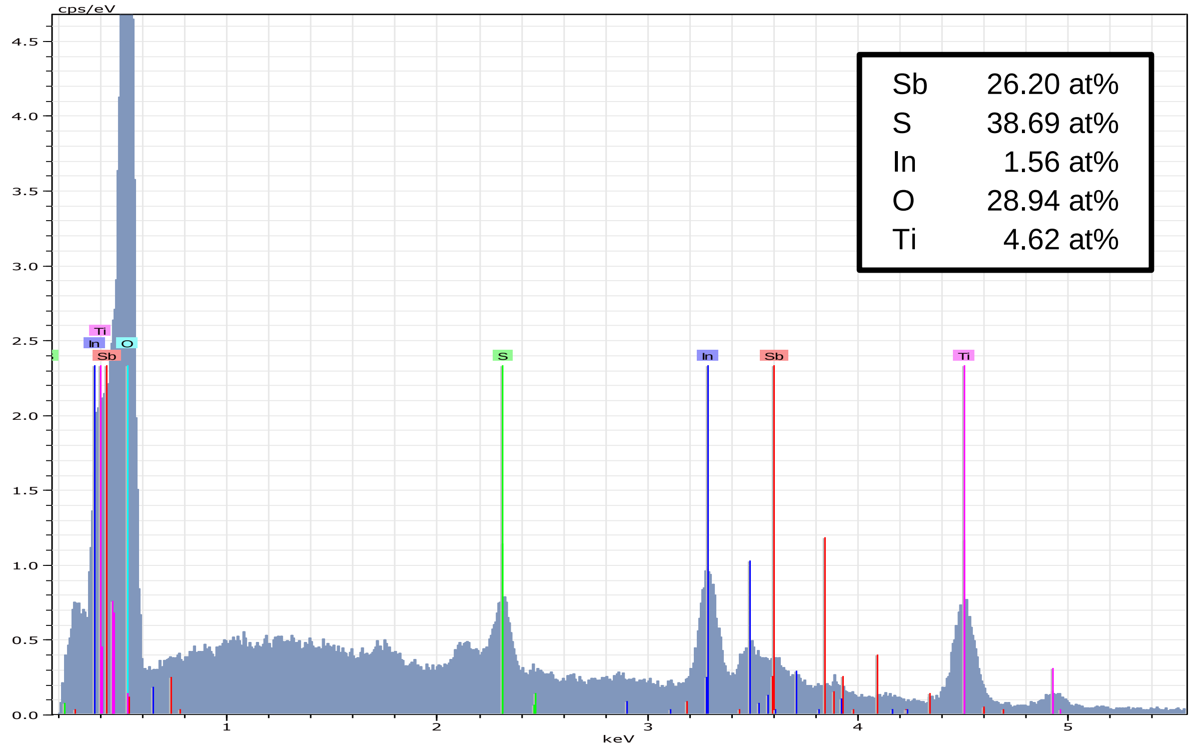

Figure S1: EDX spectrum of a glass/ITO/TiO_2/100 nm Sb_2S_3 sample after the Sb_2S_3 vacuum annealing step.

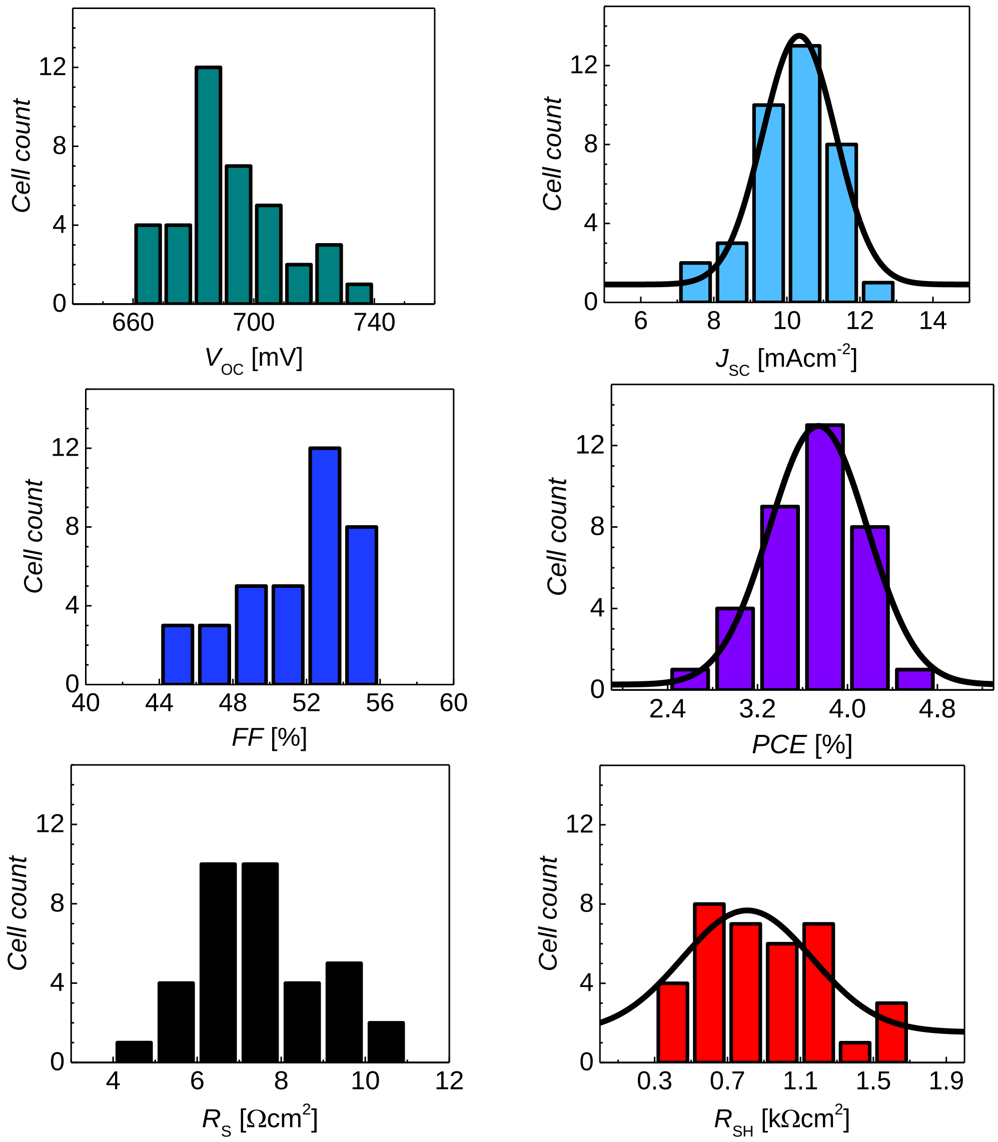

Figure S2: Distribution of V_{OC}, J_{SC}, FF, PCE, R_S, and R_{SH} in fabricated cells. Cell area 0.071 cm^2, measured at AM 1.5G, 100 mW cm^{-2}. The black lines enveloping J_{SC}, R_{SH}, and PCE are the result of Gaussian fitting, assuming normal distribution.

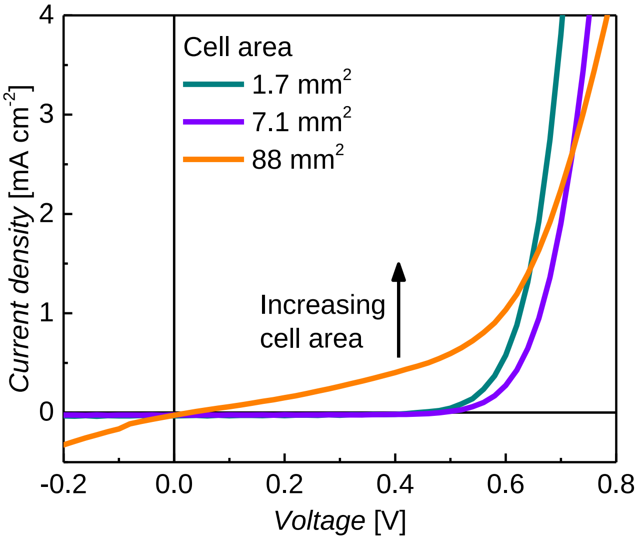

Figure S3: *J*–*V* scans in dark of solar cells with ≈100 nm Sb_2S_3, as a function of cell area.

Table S2: Photoconversion parameters[a] of a small and a large solar cell as a function of storage time.

Active area	Storage time	V_{OC}	J_{SC}	FF	PCE	R_S	R_{SH}
[mm^2]	[days]	[mV]	[mA cm^{-2}]	[%]	[%]	[Ω cm^2]	[kΩ cm^2]
7.1	0	677	9.9	52	3.4	6.1	1.4
7.1	90	703	7.6	51	2.7	5.8	0.8
7.1	120	709	7.6	48	2.6	6.5	0.5
7.1	230	724	5.2	47	1.7	5.8	0.5
88	0	648	14.5	31	2.94	24.6	232
88	14	682	14.5	33	3.22	26.2	195
88	363	686	9.4	35	2.23	41.6	233

[a]Sb_2S_3 layer thickness ≈ 100 nm. Measurement conditions: 100 mW cm^{-2}, AM 1.5G, cell area 0.071 cm^2.

References

1. Jiang, C.; Tang, R.; Wang, X.; Ju, H.; Chen, G.; Chen, T. *Solar RRL* **2019**, *3*, 1800272.
2. Zhang, L.; Wu, C.; Liu, W.; Yang, S.; Wang, M.; Chen, T.; Zhu, C. *J. Mater. Chem. A* **2018**, *6*, 21320–21326.
3. Zhang, L.; Jiang, C.; Wu, C.; Ju, H.; Jiang, G.; Liu, W.; Zhu, C.; Chen, T. *ACS Appl. Mater. Interfaces* **2018**, *10*, 27098–27105.
4. Jin, X.; Yuan, Y.; Jiang, C.; Ju, H.; Jiang, G.; Liu, W.; Zhu, C.; Chen, T. *Sol. Energy Mater. Sol. Cells* **2018**, *185*, 542–548.
5. Tang, R.; Wang, X.; Jiang, C.; Li, S.; Liu, W.; Ju, H.; Yang, S.; Zhu, C.; Chen, T. *ACS Appl. Mater. Interfaces* **2018**, *10*, 30314–30321.
6. Kaienburg, P.; Klingebiel, B.; Kirchartz, T. *Beilstein J. Nanotechnol.* **2018**, *9*, 2114–2124.
7. Lan, C.; Liang, G.; Lan, H.; Peng, H.; Su, Z.; Zhang, D.; Sun, H.; Luo, J.; Fan, P. *Phys. Status Solidi RRL* **2018**, *12*, 1800025.
8. Ye, Q.; Xu, Y.; Chen, W.; Yang, S.; Zhu, J.; Weng, J. *Appl. Surf. Sci.* **2018**, *440*, 294–299.
9. Wang, W.; Strössner, F.; Zimmermann, E.; Schmidt-Mende, L. *Sol. Energy Mater. Sol. Cells* **2017**, *172*, 335–340.
10. Kärber, E.; Katerski, A.; Oja Acik, I.; Mere, A.; Mikli, V.; Krunks, M. *Beilstein J. Nanotechnol.* **2016**, *7*, 1662–1673.
11. Zimmermann, E.; Pfadler, T.; Kalb, J.; Dorman, J. A.; Sommer, D.; Hahn, G.; Weickert, J.; Schmidt-Mende, L. *Adv. Sci. (Weinheim, Ger.)* **2015**, *2*, 1500059.
12. Kim, D.-H.; Lee, S.-J.; Park, M. S.; Kang, J.-K.; Heo, J. H.; Im, S. H.; Sung, S.-J. *Nanoscale* **2014**, *6*, 14549–14554.
